# Infusions of Digitalis in Heart Cases

**Published:** 1909-01

**Authors:** 


### Infusions of Digitalis in Heart Cases.

[*The Hospital*, December, 1908.]

THERE is a general consensus of opinion amongst those who have had most experience of the various preparations of digitalis that, although the tincture, particularly the physiologically standardised tincture, is very good indeed, the best results of digitalis action are afforded by the infusion. The difficulty is that the infusion will not keep for any length of time; it has therefore to be made up freshly at short intervals, and in country practice this means that the medical man needs to make it for himself. The following notes upon the effects of heat upon the infusion, the use of chloroform in preserving it for considerable periods, and the addition of carbonates for the same purpose, are points of practical importance. It must be assumed, of course, that the digitalis leaves have been carefully selected, gathered, and dried.

The usual method of preparing infusions is to heat to boiling-point and to keep simmering. Bokay considers it wrong to prepare infusions of digitalis by heating. The active glucoside in the leaves becomes partly decomposed when heated. The best way of making the infusion is to macerate the digitalis leaves in cold, or only moderately warm water for at least three hours. Some observers go so far as to say that the human stomach should extract the glucoside for itself, and that therefore the powdered digitalis leaves should be given as such as in the pilula hydrargyri diuretica of some hospital pharmacopeia.

When digitalis treatment is to be prolonged, and it is desired to employ the home-made infusion, it becomes a matter of importance to make the preparation keep good for as long as possible. Chloroform, like chloretone (acetone chloroform), has often been used for purposes of preservation, and it is found to answer fairly well in the case of digitalis infusions. The following recipe is one of Stepp's:—

R Digitalis foliarum ................ ʒj.

Aquam ad ........................ Oj.

Fiat infusum. Add chloroformi ...... ʒiss.

A dessertspoonful of this may be given every two hours, or a larger dose at longer intervals, the dosage and its continuance being controlled by observations of the pulse, the urine, and so forth, as when the tincture is employed. The beneficial effects of the infusion are not likely to appear sooner than the third or fourth day, but in some cases they are remarkably good.

It has been suggested that a small quantity of sodium carbonate should be added to the infusion of digitalis in order to neutralise the vegetable acids it contains. The addition seems to lengthen the time during which the infusion will keep good without other preservative to several days. Focke's prescription is as follows:

℞ Infus. Fol. Digital. Titr...℥iss. to Oj.
Sodii Carbonatis ..........gr. j.

Sig. : A teaspoonful to be taken every three hours,

